# Role of cell surface proteins and toll-like receptors in the pathogenesis of *Streptococcus pneumoniae*

**DOI:** 10.22038/IJBMS.2023.72584.15791

**Published:** 2024

**Authors:** Nurul Fathiyah Zaipul Anuar, Mohd Nasir Mohd Desa, Jamal Hussaini, Eng Hwa Wong, Vanitha Mariappan, Kumutha Malar Vellasamy, Navindra Kumari Palanisamy

**Affiliations:** 1 Institute of Medical Molecular Biotechnology (IMMB), Faculty of Medicine, Universiti Teknologi MARA (UiTM), Sungai Buloh Campus, Selangor, Malaysia; 2 Department of Biomedical Sciences, Faculty of Medicine and Health Sciences, Universiti Putra Malaysia, (UPM), Serdang, Selangor, Malaysia; 3 Department of Medical Microbiology & Parasitology, Faculty of Medicine, Universiti Teknologi MARA (UiTM), Sungai Buloh Campus, Selangor, Malaysia; 4 Institute of Pathology, Laboratory and Forensic Medicine (I-PPerForM), Universiti Teknologi MARA (UiTM), Sungai Buloh Campus, Jalan Hospital, Sungai Buloh, Selangor, Malaysia; 5 School of Medicine, Taylor’s University Lakeside Campus, Subang Jaya, Selangor, Malaysia; 6 Center for Toxicology and Health Risk Studies, Faculty of Health Sciences, Universiti Kebangsaan Malaysia, Jalan Raja Muda Abdul Aziz, Kuala Lumpur, Malaysia; 7 Department of Medical Microbiology, Faculty of Medicine, Universiti Malaya, Kuala Lumpur, Malaysia

**Keywords:** A549 cell line, Cell wall, Serotype, Streptococcus pneumoniae, Surface proteins, Toll-like receptors

## Abstract

**Objective(s):**

Pneumococcal cell wall (PCW) is an inflammatory component in *Streptococcus pneumoniae*. The cell surface proteins and the toll-like receptors (TLR) signaling response were investigated in the human lung epithelial (A549) cells inoculated with PCW of different serotypes.

**Materials and Methods:**

The presence of genes encoding these proteins was determined using polymerase chain reaction (PCR). The structure of the cell walls was analyzed by proton nuclear magnetic resonance (1H NMR). The A549 cell line was challenged with PCW extracts of different serotypes. RNA from the infected host cells was extracted and tested against a total of 84 genes associated with TLR signaling pathways (TLR 1-6 and 10) using RT2 Profiler PCR Array.

**Results:**

Cell surface proteins; *ply*, *lytA*, *nanA*, *nanB*, and *cbpD* genes were present in all serotypes. The distribution and structure of surface protein genes suggest behavioral changes in the molecules, contributing to the resilience of the strains to antibiotic treatment.

**Conclusion:**

TLR2 showed the highest expression, while serotypes 1, 3, and 5 induced higher TNFα and IL-1α, suggesting to be more immunogenic than the other strains tested

## Introduction

Pneumococci or *Streptococcus pneumoniae* is an alpha-hemolytic gram-positive bacterium that primarily affects children and causes otitis media, meningitis, pneumonia, and bacteremia. It has the capacity to circumvent host defense mechanisms and cause systemic infections including sepsis and meningitis. Every year, pneumococcal infections claim the lives of over a million children in underdeveloped countries (1). Despite the availability of pneumococcal conjugate vaccinations, two-thirds of all instances of meningitis are still caused by *S. pneumoniae* (2). The World Health Organisation has identified *S. pneumoniae* as one of the antibiotic-resistant “priority pathogens” (3), and antibiotic selective pressure has so far resulted in the creation of resistant pneumococcal clones. Pneumococcal conjugate vaccines are effective tools against antibiotic resistance, according to data from active bacterial core surveillance collected by the Centres for Disease Control and Prevention between 2009 and 2013 (4). However, these vaccinations also cause selective pressure, and *S. pneumoniae* non-vaccine serotypes are spreading globally (5, 6).

In order to cause infection,* S. pneumoniae* must overcome host immunity and reproduce in the host following colonization. The proteins on the cell surface have important functions as adhesins and/or anti-phagocytic agents. The cell wall anchoring motif LPXTG (7) and the choline-binding repeats engaging with pneumococcal phosphorylcholine (8) are two different types of motifs for pneumococcal cell surface localization. In contrast to LPXTG-anchored proteins, which are covalently connected to the cell wall, choline-binding proteins (CBPs) are located localized on the pneumococcal cell wall (PCW) via the phosphorylcholine moiety of teichoic acids. Through their interactions with host factors, a number of LPXTG-anchored proteins and CBPs aid in the adherence to host epithelial cells (7-10). By restricting complement deposition or by impeding phagocytosis, pneumococcal cell surface proteins also aid in bacterial survival (8, 11-14). 

Using pattern recognition receptors such as the toll-like receptors (TLRs), nucleotide oligomerization domain-like receptors, and retinoic acid-inducible gene-I-like receptors, the host recognizes *S. pneumoniae* in response to the pathogens and controls immune responses (15). TLRs on the host are pattern recognition receptors (PRR) that recognize the molecular elements of infections and activate the host’s immune system. As soon as it is turned on, PRR controls the synthesis of antimicrobial peptides (AMPs) and inflammatory reactions such as TNF, IL-1, IL-6, and IFN (15). The TLR family has 10 members in humans (TLR1-10): TLR1, 2, 4, 5, and 6, which are located in the plasma membrane on the cell surface, and TLR3, 7, 8, and 9, which are located in endosomes (16). The key mechanism of innate immune response to pneumococcal infections is via TLR recognition of the PRR which leads to an inflammatory response. However, in certain circumstances, these host immune reactions can lead to excessive inflammation, which can lead to complications such as neurologic damage, septic shock, and acute lung injury (17). Understanding the mechanisms behind the inflammatory response to *S. pneumoniae *may provide insight into the etiology of pneumococcal infections and help to pinpoint potential targets for new treatments.

In this study, we aimed to determine the cell surface proteins and TLR signaling response in the A549 cells inoculated with PCW of different serotypes in order to investigate the distribution of cell surface proteins which includes autolysin* (lyt*A, *psp*A, *cbp*A, *cbp*D, *cbp*E, *cbp*F, *cbp*G, *cbp*I, and *cbp*J), neuraminidases (*nanA* and *nanB*), and pneumolysin (*ply*) of S.* pneumoniae* from different serotypes. In addition, proton nuclear magnetic resonance (NMR) was used to explore the structural analysis of the cell wall of different serotypes of *S. pneumoniae*. The immune responses of these strains were further investigated by the elucidation of the TLR signaling response in A549 cells inoculated with PCW. The variation in the structure, presence of the genes responsible as surface proteins, as well as the TLR responses in host cells in relation to the different serotypes would give a better understanding of the immune response during pneumococcal infection.

## Materials and Methods

***Bacterial strains and culture conditions***

Six pneumococcal isolates of different serotypes 1, 3, 5, 19F, 23F, and 14 were obtained from American Type Culture Collection (ATCC). All strains were cultivated on tryptic soy agar (TSA) media (5% sheep blood included) and incubated for 16 to 18 hr at 37 °C with 5% CO_2_. The bacteria colonies were then cultivated until the mid-log phase in subculture on Todd Hewitt broth (THB) (BD, USA).

***Antibiotic susceptibility test***

Antibiotic susceptibility testing of the strains to β-lactams, macrolide, and fluoroquinolones, namely, penicillin (PEN), erythromycin (ERY), ciprofloxacin (CIP), levofloxacin (LVX), and cefotaxime (CTX) was investigated using disc diffusion method (Oxoid, USA). The zone diameter measurement follows the interpretive standards for *S. pneumoniae *as recommended by the Clinical and Laboratory Institute (CLSI) 2023 (18). 

***Identification of bacterial isolates***

*Bile solubility testing*

A suspension of the bacteria was added into 1.0 ml of 0.9% NaCl. A few drops of 2% sodium deoxycholate (bile salts) (Sigma, USA) were added to the suspension. The suspension was then incubated for 3 hr at 37 °C. The test tube was examined for clearing or loss of turbidity.

***Susceptibility to ethylhydrocupreine (Optochin)***

Three or four isolated colonies were suspended in 0.9% NaCl and adjusted to the concentration equivalent to 0.5 McFarland. Each of the isolated colonies was lawned onto TSA supplemented with 5% sheep blood agar to obtain confluent growth. The ethylhydrocupreine (optochin) disk was placed at the center of the plate and gently pressed onto the surface of the agar with sterile forceps. The plates were incubated for 18–24 hr at 37 °C in the presence of 5% CO_2_. The diameter of the inhibition zone was measured.

***DNA extraction for the PCR reaction***

Bacterial colonies were suspended in 1 ml phosphate-buffered saline (PBS). The suspension was centrifuged at 18,000 g for 5 min. The pellet was resuspended in 100 µl of 10 mM Tris-HCl pH 8.0. The bacterial suspension was then vortexed and heated at 100 °C for 10 min. The suspension was then centrifuged for 5 min at 15,000 g. For later usage, the DNA extraction supernatant was collected and kept at -20 °C.

*Serotyping of S. pneumoniae isolates using PCR*

The polymerase chain reaction (PCR) protocol and primers used in this investigation were modified versions of those originally developed by Pai *et al*. (2006) (19). Each reaction consists of four primer pairs that target four different serotypes and one primer pair that serves as an internal positive control and targets the* cpsA* locus, which is present in all pneumococci (20-24).

*Identification and screening of virulence genes in S. pneumoniae using PCR*

The presence of *lyt*A, *ply*, *psp*A, *nanA, nanB*, *cbp*A, *cbp*D, *cbp*E, *cbp*F, *cbp*G, *cbp*I, and *cbp*J was investigated using PCR. The primer sequences are shown in Table 1. PCR was performed in a final volume of 25 µl. Each mixture consists of 1X PCR buffer (GeneAll, Korea), 1.0 U of Taq DNA polymerase (GeneAll, Korea), 0.2 mM dNTP, 1.0 µl DNA extract, and 0.2 µM primers. The condition for PCR amplification was as follows: initial denaturation at 94 °C for 3 min followed by 25 amplification cycles of the following parameters: denaturation at 94 °C for 1 min, annealing at 52 °C for 1 min and extension at 72 °C for 1 min 30 sec and final extension at 72 °C for 10 min. The PCR products were electrophoresed on 2% agarose gel in 1X TBE buffer (Sigma-Aldrich, US), at 90 V for 1 hr. Gels were stained using SYBR Green I and visualized using Gel Doc^TM^ XR+ Imaging System (BioRad). 

*Cell wall extraction*

A previously described protocol was adapted for the PCW isolation method (25). The bacteria were cultured in 2 L of THB and then pelleted by centrifugation at 7,500 g and 4 °C. The pellet was dissolved in 40 ml of ice-cold 50 mM Tris-HCl (pH 7). Then, 150 ml of boiling 5% sodium dodecyl sulfate (SDS) (Biobasic, Canada) were added to the bacterial suspension dropwise and heated for 30 min at 95 °C. Following that, the bacterial suspension underwent a 45-min, 130,000-g ultracentrifugation at 25 °C. In order to remove all traces of SDS, the pellet was washed twice with 30 ml of 1 M NaCl and then once more with deionized water. The pellet was reconstituted in 2 ml of dH_2_O with the addition of 1/3 volume of acid-washed glass beads. The cells were then vortexed with glass beads to disrupt the cell walls. The supernatant was obtained following centrifugation at 1,000 g for 5 min. The supernatant was then subjected to a second, 45-min, 130,000-g centrifugation at 25 °C. The obtained pellet was resuspended in 20 ml of 20 mM MgSO_4_ in 100 mM Tris-HCl (pH 7.5). To the suspension was added with 10 g/ml DNase A and 50 g/ml RNase I, which was then agitated at 37 °C for two hours. Then, 100 g/ml of trypsin and 10 mM CaCl_2_ were added, and the mixture was then incubated at 37 °C overnight. Subsequently, 1% SDS final concentration was added, and the enzymes were inactivated by incubating the mixture at 80 °C for 15 min. The cell wall was separated by ultracentrifugation at 130,000 g for 45 min at 25 °C. After that, it was resuspended in 20 ml of 8 M LiCl and heated for 15 min at 37 °C. The pellet was centrifuged once again, redissolved in 10 mM EDTA pH 7, and then incubated for 15 min at 37 °C. A combination of distilled water, acetone, and distilled water was used to clean the cell wall. The cell pellet was then reconstituted in 2 ml of distilled water and kept at -80 °C for storage.

*Nuclear magnetic resonance spectroscopy*

The bacterial cell wall was dissolved in deuterium oxide. The proton NMR spectrum (1H NMR) was obtained using this solution (26). Using sample tubes with a 5 mm outer diameter, the proton NMR spectra were run on a Bruker Ascend 600 spectrometer at 600 MHz and a sample temperature of 300 K. Using TopSpin 2.1, peaks were identified and examined.

*A549 cells infected with pneumococcal cell wall*

The cell stimulation assay based on Hausler *et al*. (2002) was performed with slight modifications (27). The A549 cells seeded in six-well tissue culture plates at 2 x 10^5^ cells/well containing 2 ml of complete growth media (Dulbecco’s Modified Eagle Medium (DMEM) (Gibco, US)) supplemented with 10% fetal bovine serum (FBS) (Gibco, US) and 2% penicillin-streptomycin (Gibco, US) were allowed to adhere overnight. Upon reaching 90% confluency, the cells were washed twice with PBS. DMEM without antibiotics and FBS were added into each well and the A549 cells were then challenged with 1x10^7^/ml of the bacterial cell wall. Following that, the infected cell monolayer was incubated for 20 hr at 37 °C in the presence of 5% CO_2_. The monolayer cells were detached from the well plate using Accutase (Gibco, USA). After adding full DMEM medium, the cells were transferred to a 15 ml centrifuge tube, spun at 300 g for 5 min, and the resulting pellet was utilized to extract RNA. 

*Host cell RNA extraction after infection*

Using the RNeasy Mini Kit (Qiagen, USA), RNA from the host cells was extracted in accordance with the manufacturer’s instructions. On a 1.2% agarose gel in 1X Tris-borate-EDTA (TBE) solution, the integrity of the RNA was assessed. Using a Nanodrop ND-1000 spectrophotometer (Thermo Fisher, US), the purity of the RNA was evaluated. For the cDNA synthesis, RT2 First Strand Kit (Qiagen, USA) was obtained according to the manufacturer’s protocol.

*Toll-like receptor signaling pathway PCR array*

The expression of 84 genes essential for TLR-mediated signal transduction and immunological responses was profiled using the human TLR signaling pathway PCR array (Qiagen, USA). The PCR array is a collection of real-time PCR experiments with optimized primers on 96-well plates. Each array has PCR controls for housekeeping genes, general PCR performance, and genomic DNA contamination. A CFX96TM Real-Time PCR (Bio-Rad, US) was used to conduct the RT^2^ Profiler PCR Array. The following steps were taken to mix the PCR component parts: 1248 µl of RNase-free water, 102 µl of cDNA synthesis, and 1350 µl of 2X RT^2^ SYBR Green Mastermix. Then, each well of the 96-well plate RT^2^ Profiler PCR Array received 25 µl of the PCR component mixture. The plate was centrifuged at 1000 g for 1 min to remove bubbles after being tightly sealed with optical thin-wall eight cap strips. The CFX96TM Real-Time PCR (Bio-Rad, US) equipment was then filled with the RT^2^ Profiler PCR Array plate, and the procedure was launched. One cycle of 95 °C for 10 min, 40 cycles of 95 °C for 15 sec, 60 °C for 1 min (the ramp rate from 95 °C to 60 °C is 1 °C per sec), one cycle of 95 °C for 10 sec, and then an analysis of the melt curve from 65 °C to 95 °C with an increase in temperature of 0.5 °C make up the cycling condition for 5 sec.

***Data analysis***

Differential fold-change in gene expression was determined using the 2^-∆∆Ct^ method. Gene expression data from RT^2^ Profiler PCR Arrays were analyzed using Qiagen’s data analysis tool. Two-fold gene expression (2≥ or ≤2) was considered up-regulated and down-regulated, respectively. 

## Results

The strains were identified as *S. pneumoniae* using standard detection techniques. All strains displayed bile solubility and seemed to be ethylhydrocupreine susceptible. Using multiplex PCR with a capsule gene as an internal control, the serotypes were identified by targeting different serotypes. Serotypes 1, 3, 5, 19F, 23F, and 14 were identified, accordingly. 

The strains’ resistance to β-lactams, macrolides, and fluoroquinolone antibiotics was examined (Table 2). While serotype 23F bacteria were solely resistant to ERY, serotype 14 seemed to be resistant to both PEN and ERY. All of the other tested antimicrobials were shown to be effective against the strains of the other serotypes. The variation of virulence genes in relation to capsular properties was observed in *S. pneumoniae* of different serotypes. The amplifications of these virulence genes are summarized in Table 3. 

All tested pneumococcal serotypes, except serotypes 3 and 14 had positive amplification of *psp*A. We postulate that serotypes 3 and 14, which did not harbor the *psp*A gene, may have attenuated virulence. Surface protein, *cbp*E was amplified in serotypes 5, 19F, 23F, and 14, whereas *cbp*A gene was amplified only in serotype 19F. Pneumococcal neuraminidase, *nanA *and *nanB,* were detected in all six serotypes. Among the CBPs, *cbpG* and *cbpI* were not detected in any of the strains. The *cbpD* gene was detected in all serotypes. Additionally, *cbpF* was not detected in serotype 5, while *cbpJ* was detected in serotypes 19F, 23F, and 14.

In order to identify the structural variations, metabolites were identified by comparing with the published study by Kumari *et al*., (28) and with the comparison of the reported NMR database from the BioMagResBank (BMRB) (www.bmrb.wisc.edu). When comparing all of the serotypes using NMR, several peaks were found at chemical shifts ranging from 1.06 ppm to 3.53 ppm. The chemical shift of the typical proton NMR spectra of the various serotypes was comparable, but the peak’s intensity and frequency varied. All of the examined serotypes had chemical shifts at 1.06, 2.12, 3.25, and 3.53 ppm, but the strain of serotype 14 which was resistant to PEN and ERY had an extra peak at 2.62 ppm (Table 4).

**Figure 1:**
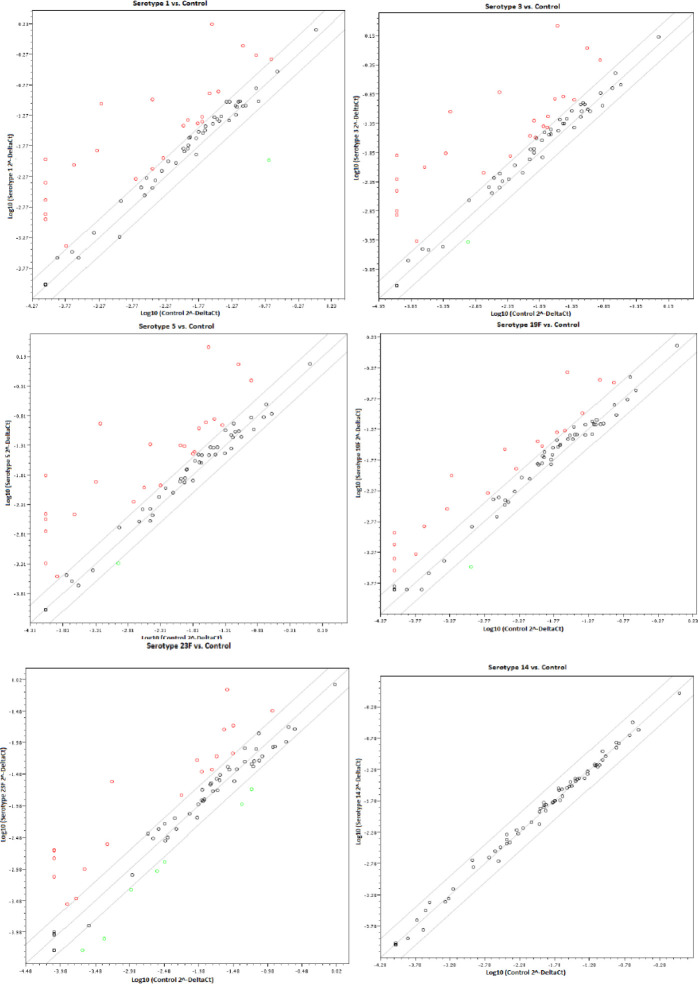
Scatter plots showing the regulation of all 84 gene expression profiles of different serotypes

In the host immune response studies, the activation of TLR triggered the activation of pro-inflammatory cytokines and the up-regulation of costimulatory molecules. The expression levels of 84 genes that were detected showed distinct regulation patterns across A549 cells infected with PCW of different serotypes. Mostly, genes observed in this study show increasing expression compared to uninfected A549 cells (control). The stimulation of A549 cells with PCW was also found to trigger the release of cytokine, chemokine, and other signaling genes. The PCR Array identified 24 up-regulated genes (≥2 fold) and a down-regulated gene in the infected A549 cells with the cell wall of serotypes 1, 3, and 5. A total of 19 genes were up-regulated, and one gene was down-regulated following infection with the cell wall of serotype 19F. The stimulation with the cell wall of serotype 23F identified 19 down-regulated genes and 8 down-regulated genes. However, the expression of genes in cells infected with a strain of serotype 14 showed unaffected gene expressions. The relative expression levels for each gene in the infected A549 cells and uninfected cells are plotted against each other in the scatter plot (Figure 1). 

**Figure 2:**
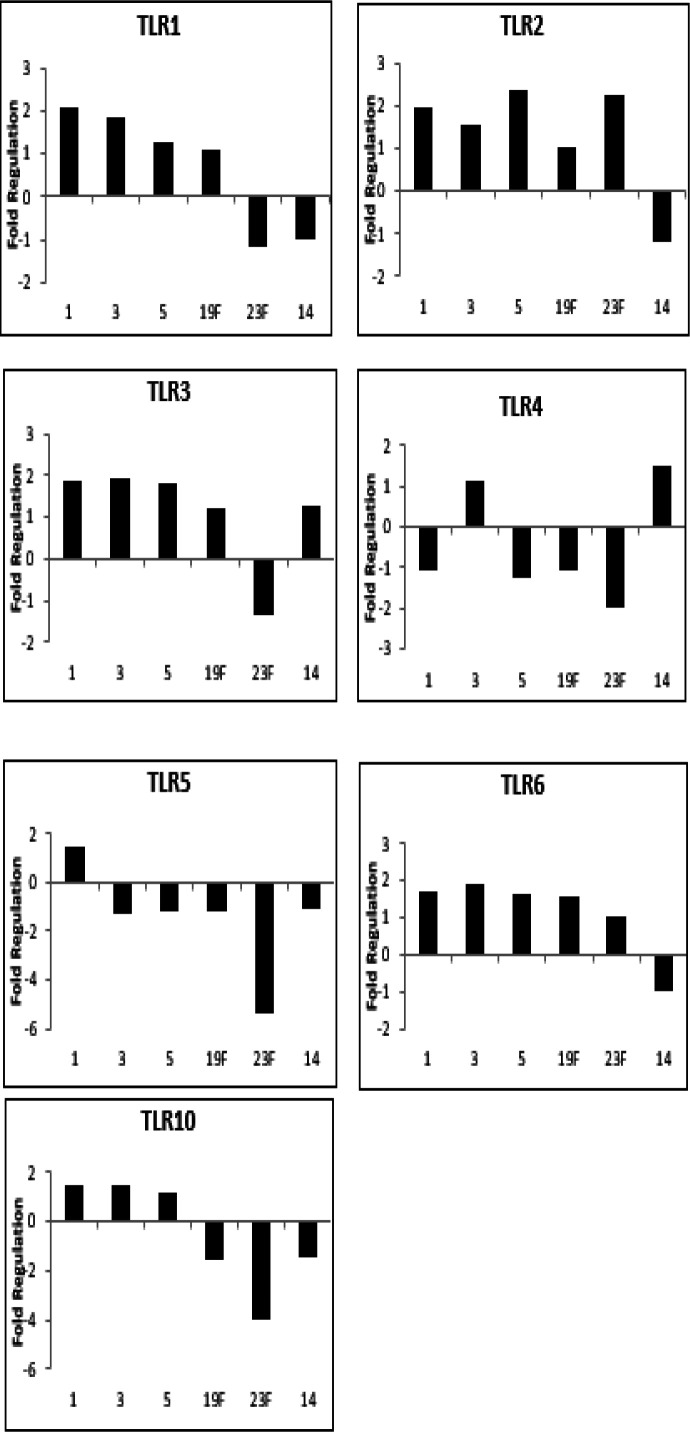
Differential expression of Toll-like receptors in A549 human lung epithelial cells compared to uninfected cells

We also found that seven out of 10 TLR receptors were found differentially regulated, while the remaining TLR receptors were undetermined. The receptors that showed distinct regulation were TLR1-6 and TLR10, while the receptors that were not expressed were TLR7-9. TLRs showed differential expression across various pneumococcal serotypes as shown in Figure 2. 

**Figure 3:**
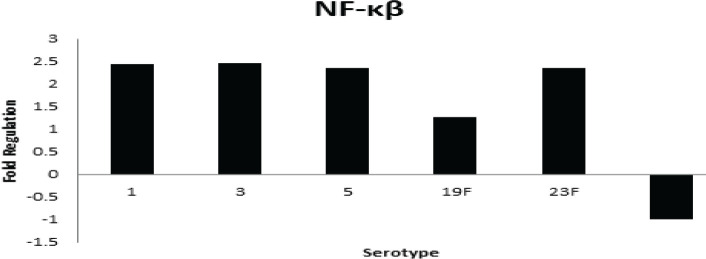
Differential expression of NF-κβ in infected A549 human lung epithelial cells compared to uninfected cells

One of the downstream signaling pathways of TLR is the nuclear factor of kappa light polypeptide gene enhancer in B-cells 1 (NF-κβ) signaling. PCW modulated A549 cells which subsequently increased NF-κB expression (Figure 3). When compared with different serotypes, serotypes 1, 3, 5, and 23F showed up-regulated expression (≥2) 6 compared to uninfected cells, whereas serotype 14 showed down-regulated gene expression. The level of NF-κB activation is associated with the virulence of the infecting organism. This suggests strains of serotypes 1, 3, 5, and 23F to be more invasive compared to serotype 14. Hence, the different abilities to cause diseases among pneumococcal serotypes is explained. 

**Figure 4:**
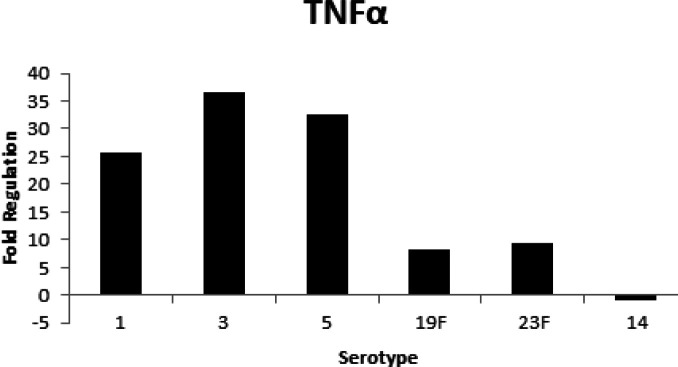
Activation of TNFα in human A549 lung epithelial cells after stimulation by pneumococcal cell wall

Consequently, the activation of NF-κB induces the production of proinflammatory cytokines like tumor necrosis factor-alpha (TNFα) and interleukin 1 (IL-1β). Infected cells showed strong induction of TNFα compared to uninfected A549 cells (Figure 4). TNFα is an important mediator in bacterial inflammation and the cellular immune response. The response to PCW demonstrated varied levels of expression of TNFα. The A549 cells that were exposed to cell wall serotypes 1, 3, 5, 19F, and 23F produced a high level of TNFα compared to unexposed cells *in vitro*. The highest expression of TNFα was observed on A549 cells infected with PCW serotypes 1, 3, and 5 with more than 25-fold up-regulation, while serotypes 19F and 23F induced 8.36 and 9.3-fold regulation, respectively. Serotype 14 showed down-regulation upon infection. 

**Table 1:** Primer sequences used in this study for the identification and screening of *Streptococcus pneumoniae *virulence genes using PCR

Primer	Forward sequence 5’-3’	Reverse sequence 5’-3’	Amplicon size (bp)	References
*lyt*A	CAACCGTACAGA ATGAAGCGG	TTATTCGTGCAATACTCGTGCG	308	[20]
*ply*	ATTTCTGTAACAGCTACCAACGA	GAATTCCCTGTCTTTTCAAAGTC	329	[21]
*psp*A	CATAGACTAGAACAAGAGCTCAAA	CTACATTATTGTTTTCTTCAGCAG	214	[22]
*nan*A	CAGTGATAGAAAAAGAAGATGTTG	ATTATTGTAAACTGCCATAGTGAA	212	[22]
*nan*B	AACTGTCCATATCTCCTATTTTTC	TATTTCTACACCTATCTCACCAGA	242	[22]
*cbp*A	GCTAATGTAGCGACTTCAGATCAA	AGCTTGGAAGAGTTTCTTCACCTA	142	[22]
*cbp*D	TGCCTGGGTGTCAAATGTAA	TTCATTGCCCCTGAACTACC	837	[23]
*cbp*E	AAGCGCCTGATTCTACAGGA	CCACTAACCAGGCACCACTT	630	[23]
*cbp*F	ATTTGCAGATGATTCTGAAGGATGG	CTTAACCCATTCACCATTCTAGTTTAAG	946	[24]
*cbp*G	TATACAGATAAGAAACAAG	ACATTAAATCCACTCA	462	[23]
*cbp*I	GGGGATGGCAGCTTTTAAAAATC	GTTTACCCATTCACCATTACC	631	[24]
*cbp*J	GGTTGTCGGCTGGCAATATATCCCGT	CCGAACCCATTCGCCATTATAGTTGAC	784	[24]

**Table 2:** Measurement of the inhibition zones of the tested pneumococcal strains against antibiotics

Note - S: susceptible, I: intermediate, R: resistant

**Table 3:** Distribution of virulence genes in the tested serotypes of *Streptococcus pneumoniae*

**ATCC strain (serotype)**					**Virulence genes**							
	***lytA***	***Ply***	***NanA***	***NanB***	***pspA***	***cbpA***	***cbpD***	***cbpE***	***cbpF***	***cbpG***	***cbpI***	***cbpJ***
**1**	*+*	*+*	*+*	*+*	*+*	*-*	*+*	*-*	*-*	*-*	*-*	*-*
**3**	*+*	*+*	*+*	*+*	*-*	*-*	*+*	*-*	*+*	*-*	*-*	*-*
**5**	*+*	*+*	*+*	*+*	*+*	*-*	*+*	*+*	*-*	*-*	*-*	*-*
**19F**	*+*	*+*	*+*	*+*	*+*	*+*	*+*	*+*	*+*	*-*	*-*	*+*
**23F**	*+*	*+*	*+*	*+*	*+*	*-*	*+*	*+*	*+*	*-*	*-*	*+*
**14**	*+*	*+*	*+*	*+*	*-*	*-*	*+*	*+*	*+*	*-*	*-*	*+*
												

**Table 4:** Chemical shift of the Proton Nuclear Magnetic Resonance (NMR) analysis for the tested serotypes of *Streptococcus pneumoniae*

The black lines indicate the two-fold change in the gene expression threshold. Fold-regulation values greater than two are indicated in red circles, while fold-regulation values less than minus two are indicated in green circles.

A549 cells were stimulated with pneumococcal cell walls of different serotypes. The expression was normalized against housekeeping genes.

**Figure 5:**
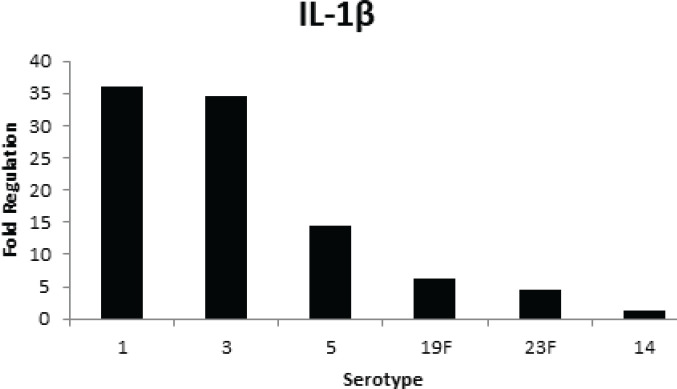
Cytokine (IL-1β) production by A549 cells stimulated with cell walls of different serotypes of* Streptococcus pneumoniae*

**Figure 6:**
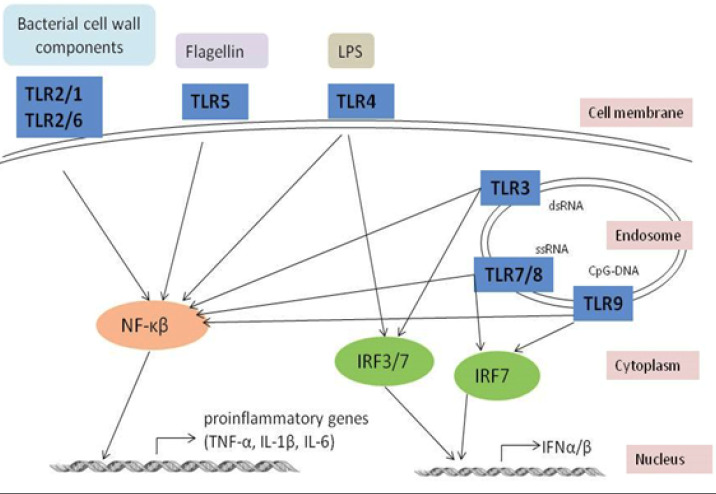
Schematic diagram depicting how Toll-like receptors (TLR) identify microbial products. The TLRs initiated signaling pathways leading to the activation of NF-κβ dependent proinflammatory gene expression and interferon regulatory factor 3/7 (IRF3/7).

## Discussion

All six serotypes used in the study demonstrated variation in the presence of gene encoding cell surface proteins. Expressions of genes encoding these genes are suggested to play a role in the pathogenesis of pneumococcal disease. *ply*, *lyt*A, *nan*A, *nan*B, and *cbpD* gene were present in all serotypes. Previous studies also documented the *ply* gene in the majority of *S. pneumoniae* isolates regardless of serotypes (29, 30). Pneumolysin causes lytic effects on many cell types and is responsible for complement activation in the absence of anti-pneumolysin antibodies and induction of proinflammatory mediators. The *lyt*A gene also plays a role in virulence, and this gene is able to digest the cell wall, which results in the release of *ply*. 

Pneumococcal neuraminidase is the most studied surface glycosyl-hydrolases due to its role in pathogenicity, as this factor is involved in the invasion of *S. pneumoniae* to the host cells. Neuraminidases increase bacterial colonization by interrupting the surfaces of competing bacteria or modifying the role of host-clearance glycoproteins. In this study, *nanA* and *nanB* genes were detected in all tested serotypes. In our previous study, *nanA* and *nanB* have an orchestrated role in causing invasive diseases in an infection model using human epithelial cell lines (29).

By interacting with the human polymeric immunoglobulin

receptor, it has been demonstrated that the* cbpA *gene promotes invasion in nasopharyngeal epithelial cells (30). Previous research established that 40–50% of the adhesion of wild-type bacteria to nasopharyngeal cells is caused by *cbpA (*31). In this investigation, the *cbpG* and *cbpI* genes were not amplified in any of the examined serotypes. Serotypes 3, 19, 23, and 14 all had *cbpF*, while serotypes 19F, 23F, and 14 all contained *cbpJ*. However, the functions are still unknown.

The peak at 2.62 ppm, which is present structurally, is indicative of carnitine, succinate, alpha-ketoglutarate, citrate, and amino acids. The presence of a peak at 2.12 ppm represents isoleucine, proline, arginine, acetate, glutamine, glutamate, N-acetyl compounds, acetylenic, and methionine, whereas metabolites discovered at 1.06 ppm represent isoleucine, leucine, valine, a methyl group, and 2-methyl glutarate. The peak for choline, phosphorylcholine, betaine, carnitine, and taurine is 3.25 ppm. Glycine and myo-inositol are represented by a peak at 3.53 ppm. One of the metabolites with a peak concentration of 2.62 ppm is an amino acid. Alanine, lysine, glutamic acid, serine, aspartic acid, and glycine are among the amino acids present in pneumococcal peptidoglycan. 

In different peptidoglycans found in the bacterial cell wall, these amino acids act as interpeptide bridges (32). The interpeptide bridges would also alter the chemical composition of the proteins. The pneumococcal autolytic enzyme needs choline residue with a peak concentration of 3.25 ppm in order to function catalytically. Due to interference with autolysin molecule adhesion to the cell wall, choline and phosphorylcholine prevent autolytic events like penicillin-induced lysis and also prevent enzymatic cell wall degradation (33). Additionally, the choline-containing teichoic acid serves as a bacteriophage receptor. Teichoic acid that contains choline is the target of and is bound by the CBPs (33, 34). These proteins that are linked to the cell wall play a crucial part in the interaction with host cells and the physiology of the cell wall. Furthermore, variations in the chemical characteristics may be indicated by differences in the cell wall composition of different serotypes. The existence of a second peak (2.62 ppm) may indicate that the cell wall structure was formed in a branching manner. Additionally, it implies that the naturally occurring cell wall, which contains penicillin-binding proteins, has been altered. These modifications would have decreased the penicillin affinity, explaining the decreased susceptibility. It might also have an impact on the organism’s ability to adapt and survive.

The connection between antibiotic resistance and cell wall structure can be seen in the relationship between the branching cell wall components and penicillin resistance. It appears that PBPs are rendered inactive by β-lactam antibiotics, which inhibit the growth of pneumococci. A branching cell wall structure may decrease the cell wall’s affinity for β-lactam medications, imparting penicillin resistance. *S. pneumoniae* PBPs have changed due to pneumococcal resistance to β-lactam. PBPs in resistant bacteria that have branched peptide chains may show a substrate preference for cell wall production. In contrast to vulnerable cell walls, which have linear chain structures, resistant cell walls have branched peptide chain architectures (28). When researching the host-pathogen immune response, immunological investigations can make use of the variations in PCW among different serotypes. 

Among the TLRs, TLR2 had a higher response to the pneumococcal cell wall. This indicates that TLR2 is important in the recognition of bacteria cell wall components. The highest expression of TLR2 expression was observed in infected cells with cell wall serotypes 5 and 23F with more than two-fold regulation, while serotype 14 showed down-regulation of genes expressed. The higher TLR2 expression in serotypes 5 and 23F suggests that these strains are more virulent, while strain 14 is less virulent. TLR4, TLR5, and TLR10 gene expression in infected A549 cells were mostly down-regulated, which means PCW is not efficient in inducing these TLR responses.

TNFα is involved in the regulation of infection related to *S. pneumoniae* serotypes. TNFα and IL-1β are important mediators of inflammation caused by gram-positive bacteria. PCW induced fold increase in IL-1β production in the A549 cell line (Figure 5). This IL-1β activity was greatly higher in serotypes 1, 3, and 5. IL-1β also showed up-regulation in serotypes 19F and 23F with 6.21 and 4.5-fold regulation, respectively. Serotype 14 demonstrated very low but detectable expression which was not highly enhanced even after infection. This suggests that IL-1β is involved in the inflammation by the PCW component and the higher IL-1β indicates more virulent strains, which means serotypes 1, 3, and 5 trigger pro-inflammatory damage compared to others. 

As compared to other studies, Gehre *et al*. (2008) (35) reported that the PCW component triggers the immune response in the cerebrospinal fluid space either in the presence of a choline cell wall or choline-free cell wall preparation. In addition, Tuomanen *et al*. (1985) (36) observed that PCW was a strong activator or triggered the meningeal inflammation *in vivo*. The study by Hausler *et al. *(2002) (27) observed that PCW is not only able to induce the production of inflammatory and microglia-active mediators from vascular endothelial cells or stimulate high microglial reactions *in vitro,* but pneumococci are capable of damaging and invading the blood-brain barrier. 

The A549 cells recognize pathogens through many types of mechanisms including TLRs. In this study, TLR1, TLR6, and TLR10 were detected when A549 cells were infected with the *S. pneumoniae* cell wall. These TLRs showed up-regulated expression which also indicates that the bacterial cell wall is efficient in inducing these TLRs responses. The study by Weber *et al*. (2003) (37) reported that HEK293 cells that lacked TLRs did not react to the pneumococcal cell wall, lipopolysaccharide (LPS), and lipopeptide (LP), while stimulation of PCW and LPS to HEK293 cells transfected with hTLR-2 was able to activate NF-κB. The finding showed the highest expression among the receptors was observed for TLR2. This postulates that among all of the TLRs, TLR2 is important in recognizing gram-positive bacteria cell walls such as peptidoglycan and lipoteichoic acid. TLR2 appears to be the most prominent among the TLR family in the immune recognition of *S. pneumoniae*. The highest TLR2 expression was observed in A549 cells infected with serotypes 5 and 23F, which means serotypes 5 and 23F are more virulent compared to the other serotypes. 

The TLRs attract several adaptor molecules and start signaling downstream of TLR, which activates the production of the NF-kβ gene (Figure 6). A variety of transcription factors, including NF-kβ and interferon regulatory factors, are activated by a particular TLR’s interaction with various combinations of adaptor proteins. The up-regulated expression of NF- kβ was observed in A549 cells infected with serotypes 1, 3, 5 19F, and 23F. This suggests that these serotypes are more virulent than serotype 14 because of the level of NF-kβ activation associated with the virulence of the infecting organism. A study by Amory-River *et al*. (2000) (38) suggested that the more invasive the infection, the degree of NF-kβ would be increased.

Another study by Bohrer *et al.* (1997) (17) observed increased NF-kβ binding activity in non-survivors compared with survivors in peripheral blood mononuclear cells. Therefore, NF-kβ is an important upstream regulator in the production of immunoregulatory and other pro-inflammatory mediators that is essential in the pathophysiology of pneumococcal pneumonia. Moreover, Heumann *et al*. (1994) (39) found that purified cell walls obtained from eight different species of gram-positive bacteria were able to induce the production of cytokines such as TNFα and IL-6 in human monocytes with the presence of 10% plasma or serum. TNFα is an effective inducer of inflammatory responses and is important during pneumococcal pneumonia for the host defense mechanism. In this study, the highest expression of TNFα was observed in serotypes 1, 3, and 5; while serotype 14 showed a low-level expression. This suggests cell wall serotypes 1, 3, and 5 are more immunogenic compared to the other serotypes because they produced higher level of TNFα. 

TNFα and IL-1β are the most important mediators of inflammation by gram-positive bacteria during infectious disease. These IL-1β and TNFα play a major role in stimulating NF- kβ activation in the pulmonary epithelium and have been observed in most cases of pneumococcal infection. The highest IL-1β expression was found in serotypes 1, 3, and 5, which means these serotypes are more pro-inflammatory compared to the other serotypes. This suggests that IL-1β is needed in inflammation by PCW on A549 cells to avoid severe diseases and death. Kafka *et al*. (2008) (40) reported that IL-1β is generally related to inflammation while IL-1α is principally involved in immunoregulation. Thus, IL-1β shows a more significant function in inducing and mediating inflammatory response compared to IL-1α. Therefore, PCW causes strong inflammation in host cells. This is demonstrated by the success of infection based on immune responses by A549 human lung epithelial cells. The *S. pneumoniae* serotype plays an important role in the occurrence of invasive disease and mortality.

## Conclusion

The finding on the distribution of cell surface proteins and their structural variation has got a role to play in the development of antibiotic resistance and indirectly has an impact on the host-pathogen interaction. The host-pathogen interaction between A549 cells with PCW showed the involvement of TLR1, TLR6, and TLR10 in modulating the immune response of the A549 cell line. Among the TLRs, TLR2 showed the highest expression. This indicates that TLR2 is important in the recognition of PCW. The up-regulated expression of NF- kβ was observed in A549 cells infected with cell wall serotype 1, 3, 5, 19F, and 23F, which means these serotypes are more immunogenic compared to serotype 14 because NF-κβ increases with the severity of infection. Serotypes 1, 3, and 5 show higher expression of TNFα and IL-1β. Serotype 23F also shows its pro-inflammatory capability by inducing TNFα and IL-1β. Therefore, the interaction between A549 cells and *S. pneumoniae* components might give a better understanding of host-pathogen interactions and immune responses. Further investigation *in vivo* would be beneficial to explore host-pathogen interaction. It is important to study the serotype prevalence of *S. pneumoniae* in Malaysia and thus aid in the vaccine development for better coverage in our setting.

## Authors’ Contributions

NK P conceived and designed the study protocol. NF ZA collected the study samples, performed the laboratory tests, and searched the literature. NF ZA, V M, and KM V carried out sample data acquisition. NK P, V M, KM V, and MN MD analyzed data and interpreted the results. NK P, MN MD, W EH, KM V, and V M supervised laboratory work and data analysis. All authors participated in drafting the article. NK P, NF ZA, and V M wrote the final manuscript. All authors have approved the final manuscript for publication.

## Conflicts of Interest

No potential conflicts of interest were reported by the author(s).
